# Preoperative Embolization in the Management of Giant Thoracic Tumors: A Case Series

**DOI:** 10.3390/jpm14101019

**Published:** 2024-09-24

**Authors:** Nicola Maria Lucarelli, Nicola Maggialetti, Giuseppe Marulli, Pierluigi Mariani, Ilaria Villanova, Alessandra Mirabile, Chiara Morelli, Angela De Palma, Amato Antonio Stabile Ianora

**Affiliations:** 1 Interdisciplinary Department of Medicine, Section of Radiology and Radiation Oncology, University of Bari “Aldo Moro”, 70124 Bari, Italy; 2 Division of Thoracic Surgery, IRCCS Humanitas Research Hospital, 20089 Milan, Italy; 3 Department of Biomedical Sciences, Humanitas University, 20090 Milan, Italy; 4 U.O.C. Radiologia, P.O. San Paolo, ASL Bari, 70123 Bari, Italy; 5 Neuroradiology Unit, Azienda Ospedaliera Consorziale Policlinico di Bari, 70124 Bari, Italy; 6 Unit of Thoracic Surgery, Department of Precision and Regenerative Medicine and Ionian Area, University of Bari “Aldo Moro”, 70124 Bari, Italy

**Keywords:** giant thoracic tumors, transcatheter arterial embolization, preoperative embolization, computed tomography, digital subtraction angiography

## Abstract

**Objectives**: The aim of this paper is to describe our experience in the embolization of hypervascular giant thoracic tumors before surgical excision. **Methods**: A single-center retrospective review of five trans-arterial preoperative embolization procedures executed between October 2020 and July 2024. Patients’ demographics, anatomical aspects, feasibility, technique, and outcomes were reviewed. **Results**: In all cases, accurate targeting and safe embolization was achieved, with satisfactory devascularization evaluated with post-procedural angiography and with minimal blood loss during subsequent surgical operation. **Conclusions**: In our experience, preoperative embolization of giant thoracic masses has been technically feasible, safe, and effective in reducing tumor vascularization, thus facilitating surgical treatment. This approach should be evaluated as an option, especially in patients with hypervascular thoracic tumors.

## 1. Introduction

Giant thoracic tumor (GTT) is a term usually designated to describe a thoracic mass that presents with dimensions of 10 cm or more on the long axis or that occupies the whole hemithorax.

These masses are relatively uncommon, accounting for 2% of thoracic tumors, and can exhibit a wide range of different histologies [1,2]. They may be characterized by rich and aberrant vasculature with multiple arterial feeders; hence, complete surgical resection is an imposing challenge. Intraoperative bleeding is one of the main safety concerns, contributing to increased morbidity, mortality, and operative time [3].

Transcatheter arterial embolization (TAE) is a well-established interventional procedure, primarily used to control hemoptysis due to the tumor’s systemic arterial supply or bleeding resulting from surgical treatment [4]. Some authors have suggested the use of preoperative embolization to limit the risk of severe intraoperative hemorrhage, identifying surgically inaccessible arterial feeders, inducing perilesional edema that facilitates dissection, and reducing tumor volume to some extent [5,6]. Thus, preoperative embolization could also lead to a significant decrease in surgical operative time.

Very few case series are available in the literature, and no guidelines have been produced regarding the role of the interventional radiologist in the management of highly vascular GTT.

We report our experience in preoperative embolization of five GTTs, reviewing the demographics, imaging features, safety, and efficacy of the angiographic procedure.

## 2. Materials and Methods

Five patients, three females and two males, with GTT were referred to our institution between October 2020 and July 2024; all underwent preoperative TAE. Their ages ranged from 32 to 72 years, with a median age of 52 years. All patients were evaluated with a contrast-enhanced Computed Tomography (CT) scan before the procedure (Siemens SOMATOM Definition AS). Regarding the angiographic technique, a right percutaneous femoral arterial access was obtained using the Seldinger technique; an aortography was then performed using a 5-Fr pigtail catheter to identify tumor-feeding arteries, followed by selective catheterization with a 5-Fr Simmons or Cobra catheter to study potentially dangerous anastomoses and anatomical variants. Superselective angiography was carried out using a 1.7-Fr or a 2.7-Fr microcatheter. Detachable microcoils and Polyvinyl Alcohol (PVA) particles (180–300 µm, 355–500 µm, 500–700 µm in size) were used as embolic agents.

All patients underwent surgical excision of the tumor within 48 h of embolization. No-one reported any side effect after the embolization.

Diagnosis was confirmed with a postoperative histological examination of the tumor. Histotypes of the masses were Schwannoma (one case), neuroendocrine lung tumor (one case), solitary fibrous tumor of the pleura (two cases), and ganglioneuroma (one case).

Case 1

**Figure 1:**
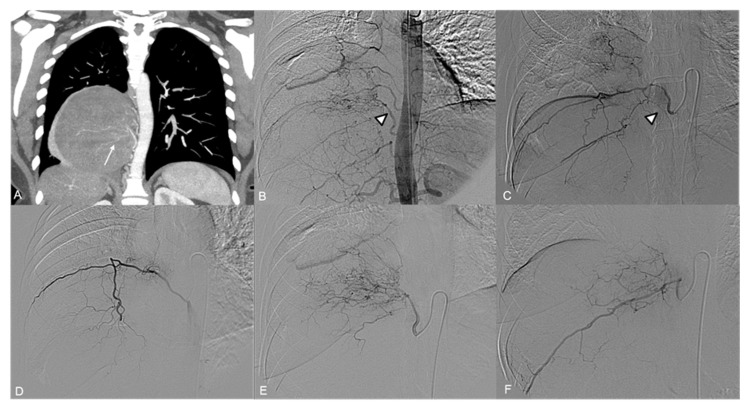
(**A**) CT angiography, coronal view: large right paravertebral thoracic mass with multiple arterial feeders (arrow). (**B**) The preliminary aortography demonstrates a rich vascularization of the tumor, mainly by branches from the VI, VIII, and X intercostal artery and an aberrant aortic branch (arrowhead). (**C**–**F**) Selective angiography of the principal arterial feeders; note a radiculomedullary artery arising from the VIII intercostal artery (arrowhead in (**C**)).

A 38-year-old (y.o.) female with thoracic pain underwent a CT examination which revealed a large hypervascular right paravertebral thoracic mass (Figure 1A).

Angiography showed multiple arterial feeders to the tumor (Figure 1B): a descending branch from the VI right intercostal artery, ascending branches from the VIII and X right intercostal arteries, and an aberrant branch originating directly from the right aspect of the thoracic aorta between the VIII and the X intercostal arteries (Figure 1D–F)

Selective catheterization of the right VIII intercostal artery demonstrated anastomoses with the IX right intercostal artery and a median descending vessel arising from the VIII intercostal artery, potentially a spinal artery (Figure 1C).

**Figure 2:**
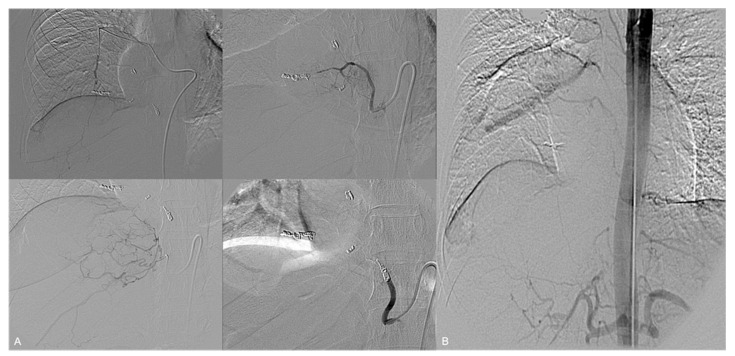
(**A**) Superselective embolization with PVA particles and microcoils. (**B**) Final aortography shows devascularization of the mass.

To prevent a distal reflux of embolic material into the spinal artery, superselective catheterization with a 1.7-Fr microcatheter was performed and then embolization was executed beyond these anastomoses by using a total amount of 5 detachable microcoils; the remaining arterial vessels to the tumor were embolized with 300–500 µm PVA particles (Figure 2).

A right postero-lateral thoracotomy was performed at the VI intercostal space.

The endothoracic mass originated from the posterior mediastinum with paravertebral extension. After lysing the adhesion, the tumor was isolated by sectioning and suturing the arterial and venous vascular peduncles.

The patient underwent neurophysiological monitoring to isolate the intercostal arteries.

Two chest drainages were placed, and the chest wall was closed by layers.

Somatosensory and motor-evoked potentials remained unchanged at the end of the procedure.

Postoperative histological examination identified the tumor as a myxoid schwannoma.

Case 2

**Figure 3:**
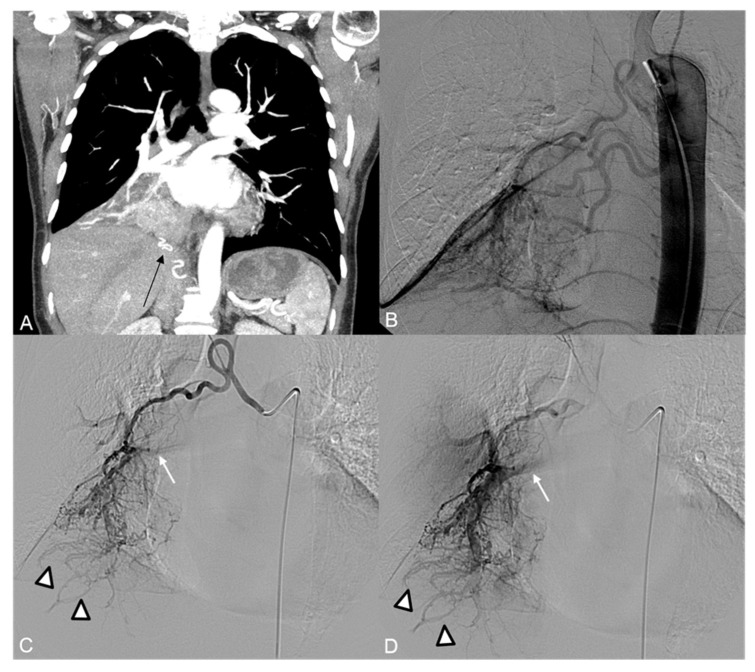
(**A**) CT Angiography, coronal view: highly vascular mass inferior to the right hilum. Hypertrophic right phrenic artery ascending to the mass (arrow). (**B**) The preliminary aortography shows the presence of three hypertrophic bronchial feeders to the tumor with intralesional fistulae between bronchial arteries and the inferior pulmonary veins. (**C**,**D**) Selective angiogram of the superior right bronchial artery in different consecutive phases shows the recruitment of enlarged bronco-pulmonary anastomoses (arrowhead), with early opacification of the inferior pulmonary vein (arrow).

A 47 y.o. female with dyspnea underwent a CT examination which revealed a hypervascular mass in the right lower lobe (Figure 3A).

Aortography showed a hypervascular lesion fed by multiple hypertrophic tortuous vessels originated by the two main right bronchial arteries. Early venous drainage in the right pulmonary veins was detected, revealing the recruitment of hypertrophied bronco-pulmonary anastomoses (Figure 3B–D).

**Figure 4:**
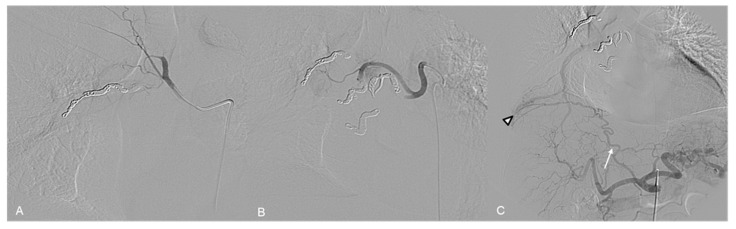
(**A**,**B**) The three bronchial feeders are superselectively microcatheterized and embolized with detachable coils. (**C**) Selective catheterization of the celiac trunk reveals hypertrophic right phrenic artery (arrow) feeding the tumor and communicating with the right inferior pulmonary vein (arrowhead).

Due to the risk of paradoxical embolization, the use of liquid embolic agents and particles was deemed unsafe. The embolization of the main bronchial arterial feeders was carried out using a total amount of 23 detachable microcoils (Figure 4A,B).

Selective catheterization of the celiac trunk and superselective catheterization of its branches demonstrated a redundant arterial supply to the tumor by a transdiaphragmatic aberrant artery originating from the right phrenic artery, which was hypertrophied and tortuous. Early drainage from these arteries into the right inferior pulmonary vein was also displayed, suggesting another probable intralesional artero-venous fistula (Figure 4C).

Proximal embolization of the right phrenic artery was avoided to prevent ischemic complications to the right hemidiaphragm.

A right postero-lateral thoracotomy was performed at the V intercostal space.

Upon opening the pleural cavity, tight adhesions between the lower lobe and the diaphragm were noted and then lysed. An anomalous, tortuous, and hypertrophic phrenic artery was identified at the pulmonary hilum and clipped upstream. The artery, vein, and bronchus adjacent to the lesion were ligated, and lymphadenectomy was performed.

Two chest drainages were placed, and the chest wall was closed by layers.

The postoperative histological examination revealed an atypical carcinoid of the lung.

Case 3

**Figure 5:**
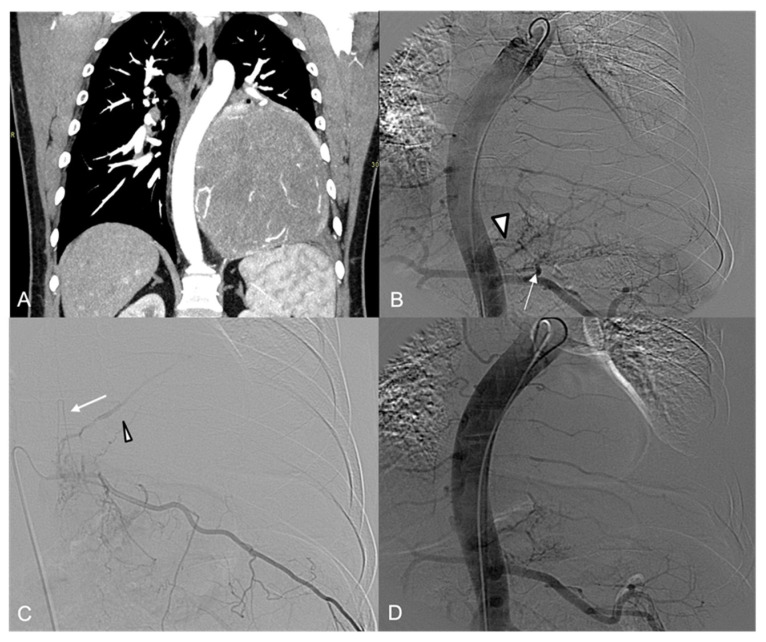
(**A**) CT Angiography, coronal view: large, highly vascular mass in the left hemithorax dislodging the thoracic aorta. (**B**) Preliminary aortography shows the rich vascularization of the mass, with branches arising mainly from the left phrenic artery (arrow) and the first left lumbar artery (arrowhead). (**C**) Selective catheterization of the X intercostal left artery shows the presence of the Adamkiewicz artery (arrow) and a minimal supply to the tumor (arrowhead). (**D**) Highly reduced tumor vascularization at the final aortography.

A 45 y.o. male with thoracic pain underwent a CT examination which revealed a hypervascular left thoracic mass (Figure 5A).

The aortography and selective angiography of the main left intercostal arteries, left bronchial artery, lumbar arteries, and celiac trunk were executed. Multiple arterial feeders were identified: branches from the VIII and X left intercostal arteries, left bronchial artery, first left lumbar artery, and left phrenic artery (Figure 5B).

Selective catheterization of the X left intercostal artery demonstrated the origin of the Adamkiewicz artery, along with a small arterial supply to the tumor (Figure 5C).

Due to its minor contribution to the tumor’s arterial supply and due to the risk of neurologic complications potentially arising from inadvertent embolization of the anterior spinal artery, the embolization of the X intercostal artery was not performed.

The remaining feeders were embolized via coaxial microcatheterization (2.7-Fr), using 355–500 µm PVA particles.

A left postero-lateral thoracotomy was performed at the VI intercostal space.

The mass occupied the lower half of the hemithorax, with possible infiltration of the diaphragm.

Costostomy removing the lateral arch of the left VII rib was performed. Part of the thickened pleura and part of the pericardial fat were also excised. The artery, vein, and bronchus for the lower lobe were ligated.

A lower lobectomy, including the tumor and the infiltrated portion of the diaphragm, was performed en bloc; the diaphragm breach was sutured.

A lymphadenectomy was conducted.

A chest drainage was placed, and the chest wall was closed by layers.

A postoperative histological examination revealed a solitary fibrous tumor of the chest wall.

Case 4

**Figure 6:**
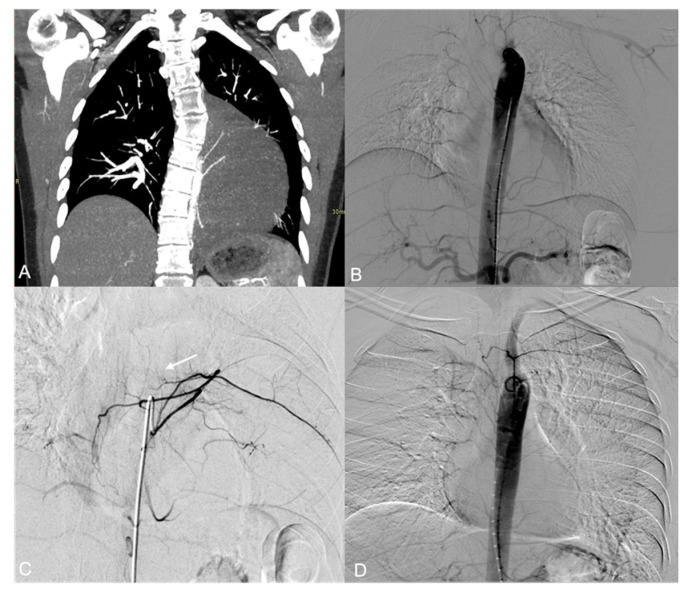
(**A**) CT Angiography, coronal view: large mass in the posterior left mediastinum dislodging arterial pedicles arising from the thoracic aorta. (**B**) Preliminary aortography shows the mass has arterial feeders arising mainly from the left intercostal arteries. (**C**) Selective angiography of the VI intercostal left artery shows the presence of medial radicular branches (arrow). (**D**) Final aortography after PVA particle embolization.

A 32 y.o. female with thoracic pain underwent a CT examination, which demonstrated hypervascular left paravertebral mass (Figure 6A).

Aortography and selective catheterization of left intercostal arteries (Figure 6B) showed arterial supply to the tumor with branches arising from the VI, VII, and VIII intercostal arteries, which were dislodged by the mass.

Proximal embolization of VII and VIII left intercostal arteries was executed via coaxial microcatheterization (2.7-Fr) using 355–500 µm PVA particles.

Superselective angiography of VI left intercostal artery revealed the presence of medial radicular branches, suspicious for spinal feeders, coursing in proximity of the intervertebral foramina (Figure 6C); therefore, embolization of this pedicle was not performed.

A left postero-lateral thoracotomy was performed at the VI intercostal space.

The endothoracic mass originated from the posterior mediastinum with paravertebral extension. The tumor was isolated by cutting and suturing the arterial and venous vascular peduncles arising from the thoracic aorta. The lesion was detached from the costovertebral sinus. The patient underwent neurophysiological monitoring to isolate the VI, VII, and VIII intercostal arteries. The lesion was separated from the costo-vertebral plane.

Two chest drainages were placed, and the chest wall was closed by layers.

Somatosensory and motor-evoked potentials remained unchanged at the end of the procedure.

Postoperative histological examination revealed a ganglioneuroma.

Case 5

**Figure 7:**
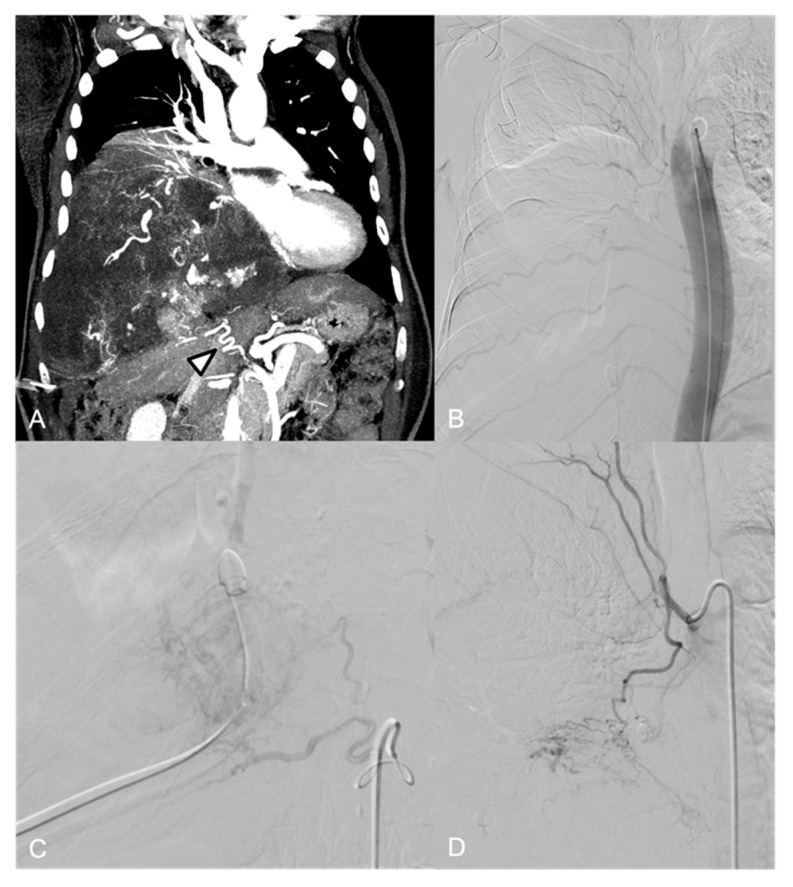
(**A**) CT Angiography, coronal view: large mass in the posterior right hemithorax. Vascular supply from the right phrenic artery arising from the abdominal aorta (arrowhead). (**B**) Preliminary aortography shows hypertrophy of right intercostal arteries. (**C**) Selective angiography of the right phrenic artery and (**D**) right intercostobronchial trunk, with their supply to the tumor.

A 72 y.o. male with severe dyspnea underwent a CT examination which demonstrated a large hypervascular right chest mass (Figure 7A).

The preliminary aortography showed the tumor’s pathological vascularization, with feeders arising from a hypertrophic right intercostobronchial trunk, right intercostal arteries from VI to XII, the first two right lumbar arteries, and the ipsilateral phrenic artery arising from the abdominal aorta (Figure 7B,C).

**Figure 8:**
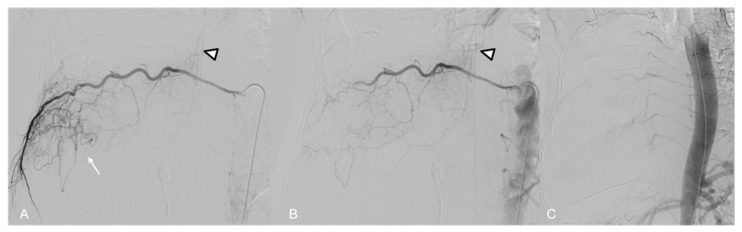
(**A**) Selective angiography of the VIII intercostal right artery demonstrates vascular supply to the tumor (arrow) and the anterior spinal artery of Adamkiewicz (arrowhead) arising from its radiculomedullary trunk. (**B**) Control after embolization demonstrates the preserved patency of the spinal artery (arrowhead). (**C**) Final aortography shows good devascularization of the tumor.

Selective catheterization of the VIII intercostal artery identified Adamkiewicz’s artery originating from its radiculo-medullary branch (Figure 8A).

After superselective microcatheterization (2.7 Fr), all feeders were embolized using 355–500 µm PVA particles.

Embolization of the right VIII intercostal artery was performed distally to prevent any proximal reflux of particles in the anterior spinal artery (ASA) (Figure 8B).

A right postero-lateral thoracotomy was performed at the VI intercostal space.

Upon opening the pleural cavity, a giant tumor of the pleura was observed, displacing the lung, hemidiaphragm, and mediastinum.

Costostomy removing the antero-lateral arch of the right VI rib and costotomy of the right VII ribs were performed, after cutting and suturing the intercostal artery.

The tumor was isolated after sectioning and suturing the arterial and venous vascular peduncles arising from the visceral pleura of the diaphragmatic surface of the lower right lobe.

All the adhesions were lysed.

Three chest drainages were placed, and the chest wall was closed by layers.

The postoperative histological examination of the tumor revealed a solitary fibrous tumor of the pleura.

## 3. Results

Embolization was performed in all patients without any perioperative complication. Satisfactory reduction in the tumor’s vascularization was obtained, as demonstrated by aortographies performed at the end of each procedure.

All patients underwent surgery within 48 h after the embolization procedure, with minimal blood loss.

## 4. Discussion

GTTs are rare entities that could be benign and malignant. Common types include germ cell tumors, sarcomas, and thymomas, while others reported in the literature include schwannoma, paraganglioma, hemangiopericytoma, hemangioendothelioma, leiomyoma, benign solitary fibrous tumor, lipoma, desmoid tumor, and Castelman disease [7,8,9,10,11].

Reported mean age of diagnosis in two studies by Shi et al. and Sunam et al., was 33 and 40 respectively [1,2].

Benign masses are usually asymptomatic in the early stages of growth because of their poor invasiveness, with symptoms emerging later due to compression of surrounding organs; consequently, they can reach considerable dimensions and can develop hypervascularization.

Malignant tumors are more often symptomatic, given their ability to invade adjacent anatomical structures and metastatize.

In our case series, we report a Schwannoma, a neuroendocrine lung tumor, two solitary fibrous tumors, and a ganglioneuroma.

Surgical resection “en bloc” is the treatment of choice in most cases; when it is not feasible, i.e., for non capsulated lesions, piecemeal excision represents a surgical alternative.

Due to the size of these masses, achieving a wide and correct surgical exposure of all vascular and neural structures often presents technical difficulties; an accidental injury to a large feeding vessel may cause massive hemorrhages, requiring extension of the operative field to rapidly achieve hemostasis, thereby increasing perioperative morbidity and mortality [3].

Preoperative imaging, in particular with CT angiography, is very useful for assessing the vascularization of the GTT; large and tortuous feeding arterial branches may derive from bronchial arteries, internal mammary artery, thyrocervical trunk, intercostal arteries, pulmonary artery, coronary arteries, and from abdominal arteries with transdiaphragmatic course [5,12,13,14,15,16,17].

Digital subtraction angiography (DSA) serves as an adjunctive diagnostic modality, identifying vascular supply to the tumor, and has an important role in the embolization of the tumor’s arterial feeding vessels before surgical treatment, as reported in the literature [5,6,13,15,18,19,20].

Compared to CT, DSA has higher sensitivity in identifying the vascular pattern of the tumor, as well as in detecting possible dangerous anastomosis with small non-expandable arteries, such as spinal arteries, and in the detection of possible hypertrophied bronco-pulmonary anastomosis [21,22].

Therefore, before proceeding with the embolization procedure, a preliminary angiography study is mandatory for assessing vascular supply of the tumor.

Transarterial embolization of the tumor minimizes intraoperative blood loss and the ischemia-induced perilesional edema makes surgical exposure and dissection relatively safer. A reduction in tumor size up to 30% has also been described for GTTs [19].

Regarding technique, non absorbable materials are generally preferred in order to achieve a better devascularization. PVA particles are among the most common embolic agents reported in the literature; smaller particles provide distal embolization at the capillary level, while particles ranging from 250 to 500 µm in diameter are used to occlude larger vessels and to prevent recruitment of small anastomoses [18]. As embolic materials, we used both detachable coils and PVA particles.

Particle injection should be performed slowly with close attention under fluoroscopy guidance to avoid the reflux of embolic material into non-target vessels [19].

A common finding during angiography of GTTs is the presence of intralesional shunts between systemic circulation (typically bronchial arteries) and pulmonary veins. Bronco-pulmonary shunts are normally present but not recruited; bronchial arteries, in fact, do not participate in alveolar gas exchanges and drain into bronchial veins tributary of the azygos venous system. In some pathological conditions (hypoxia, chronic inflammation, neoplasms), these anastomoses become more relevant in response to vascular growth factors like EGF-1 and can be responsible for recurrent hemoptysis [23,24]. In this scenario, the use of particles and liquid agents should be avoided due to the risk of paradoxical embolization; therefore, detachable microcoils offer a better level of safety. In one case in our series, angiography revealed the presence of intralesional shunts: branches from the right bronchial arteries and the right phrenic artery drained into the inferior right pulmonary vein, hence we used detachable microcoils to embolize the bronchial branches.

Another important consideration is the inadvertent embolization of spinal arteries [25]. The ASA receives radiculomedullary branches arising from the intercostal, bronchial, lumbar, vertebral, subclavian, or internal iliac artery. During embryological development, paired segmental radiculomedullary arteries arise at each vertebral body level, while in adults, the ASA is supplied by only six to eight branches. The artery of Adamkiewicz (arteria radicularis magna) is the most important of these, supplying the two lower thirds of the spinal cord, and in 75 percent of individuals it originates between D9 and D12 from the left side [26]. Because the ASA vascularizes such a large area of the spinal cord, the occlusion of a radiculomedullary artery can lead to severe neurologic complications, such as permanent complete paraplegia [27,28]. Therefore, when a “dangerous” anastomosis is detected at the preliminary angiograms, the embolization should only be executed distally to the origin of the spinal feeder, possibly using detachable microcoils and avoiding any embolic material that could determine undesired reflux in the spinal artery [19].

Nevertheless, neurologic ischemia-related complications can be verified despite the adoption of these precautions, as reported by some authors [29].

In one of our cases (case 1), after the identification of an anastomosis with a median radiculomedullary artery, we performed distal coiling of an intercostal feeder; in another case (case 3), we avoided the embolization as we visualized a communication between a tumor-feeding vessel and the Adamkiewicz artery. In a further case (case 4), embolization of the feeders was successfully executed distally in respect to the origin of the spinal arteries, carefully checking for unwanted reflux of embolic material.

Aside from generic complications related to vessel catheterism (dissection, vessel perforation, groin hematoma, etc.), other complications described in the literature are fever, chest pain, and transitory dysphagia [30].

## 5. Conclusions

This small case series suggests that preoperative arterial embolization of GTTs can be both safe and effective in reducing the tumor’s vascular burden, thereby facilitating subsequent surgical treatment. Although randomized and controlled studies on a larger scale are needed to obtain standardized indications, preoperative embolization should be considered for patients with GTTs that exhibit arterial hypervascularization at CT angiography.

## Data Availability

Data are contained within the article.
